# Modified poly(L-lysine)-based structures as novel antimicrobials for diabetic foot infections, an *in-vitro* study

**DOI:** 10.12688/hrbopenres.13380.1

**Published:** 2022-01-12

**Authors:** Alicia Grace, Robert Murphy, Aoife Dillon, Diarmuid Smith, Sally-Ann Cryan, Andreas Heise, Deirdre Fitzgerald-Hughes

**Affiliations:** 1 Department of Microbiology,, Beaumont Hospital, Dublin, D09V2N0, Ireland; 2 Department of Clinical Microbiology,, Royal College of Surgeons in Ireland (RCSI) University of Medicine and Health Sciences, The Smurfit Building, Beaumont Hospital, Dublin, D09 YD60, Ireland; 3 Department of Chemistry, Royal College of Surgeons in Ireland (RCSI) University of Medicine and Health Sciences, 123 St. Stephens Green, Dublin 2, D02 YN77, Ireland; 4 Department of Endocrinology, Diabetes Centre, Beaumont Hospital, Dublin, Ireland, D09 V2N0, Ireland; 5 SFI Advanced Materials and Bioengineering Research Centre (AMBER), Royal College of Surgeons (RCSI) University of Medicine and Health Sciences and University of Dublin, Trinity College, Dublin, Ireland; 6 SFI Centre for Research in Medical Devices (CÚRAM), Royal College of Surgeons in Ireland, University of Medicine and Health Sciences and National University of Ireland, Galway, Ireland; 7 School of Pharmacy & Biomolecular Sciences, Royal College of Surgeons in Ireland (RCSI) University of Medicine and Health Sciences, 123 Saint Stephen's Green, Dublin 2, D02 YN77, Ireland

**Keywords:** Peptide polymers; antimicrobial; diabetic foot infection; wound infection; biomaterials

## Abstract

**Background: **Wound infections occur as sequelae to skin trauma and cause significant hospitalizations, morbidity and mortality. Skin traumas arise more frequently in those with diabetes or cardiovascular disease and in these settings, may be chronic with poorer outcomes including lower limb amputation. Treatment of chronic wound infection is challenging due to antibiotic resistance and biofilm formation by bacteria including *S. aureus* and *P. aeruginosa,* which are among the most frequent causative pathogens. Managing these challenging infections requires new molecules and modalities. **Methods:** We evaluated antimicrobial and anti-biofilm activity of star-shaped poly(L-lysine) (PLL) polymers against *S. aureus* and *P. aeruginosa* strains and clinical isolates recovered from wounds including diabetic foot wounds (DFW) in a Dublin Hospital in 2019. A star-shaped PLL polypeptide series, specifically G2(8)PLL _20_, G3(16)PLL _10_, G4(32)PLL _5_ with variation in polypeptide chain length and arm-multiplicity, were compared to a linear peptide, PLL _160_ with equivalent number of lysine residues. **Results: **All PLLs, including the linear polypeptide, were bactericidal at 1μM against *S. aureus* 25923 and *P. aeruginosa* PAO1, with log reduction in colony forming units/ml between 2.7-3.6. PLL _160 _demonstrated similar killing potency against 20 *S. aureus* and five *P. aeruginosa* clinical isolates from DFW, mean log reductions: 3.04 ± 0.16 and 3.96 ± 0.82 respectively after 1 hour incubation. Potent anti-biofilm activity was demonstrated against *S. aureus* 25923 but for clinical isolates, low to moderate loss of biofilm viability was shown using PLL _160 _and G3(16)PLL _10_ at 50 μM ( *S. aureus*) and 200 μM ( *P. aeruginosa*) with high inter-isolate variability *. *In the star-shaped architecture, antimicrobial activity was retained with incorporation of 5-mer hydrophobic amino-acid modifications to the arms of the polypeptides (series G3(16)PLL _20_-coPLT _5_, G3(16)PLL _20_-coPLI _5_, G3(16)PLL _20_-coPLP _5_). **Conclusion: **These polypeptides offer structural flexibility for clinical applications and have potential for further development, particularly in the setting of diabetic foot and other chronic wound infections.

## Introduction

Chronic wound infections are common in community and healthcare settings and result in significant hospitalizations, morbidity and mortality. These infections include infected pressure sores, surgical site infections, burns and diabetic foot infections (DFI). DFIs are among the strongest predictors of lower limb amputation ^1^ and annually, more than one million people with diabetes suffer lower limb loss following failure of therapeutic interventions for DFI ^2^. Systemic antibiotic therapy is central to the range of multidisciplinary interventions for DFI. Despite the potential advantages of topical antimicrobial agents, such as targeting high concentrations directly to the site of infection and avoidance of toxic systemic effects, to date they have not been considered an effective replacement for systemic agents. However, there is a paucity of well-designed randomised controlled trials in this area ^3, 4^.

The microflora of chronic wounds is heterogenous and poly-microbial but the opportunistic pathogens, *Staphylococcus aureus* and *Pseudomonas aeruginosa* are among the most frequently isolated bacteria from these infections in humans ^5, 6^. In the setting of chronic wounds, these organisms produce biofilms, assembled communities of surface-bound cells enclosed in an extracellular matrix. In addition to resistance to phagocytosis, biofilms are recalcitrant to many antibiotics. This feature can complicate and significantly extend antibiotic treatment and may require higher antibiotic concentrations to reach the site of infection due to poor penetration. Peripheral arterial disease, if present, may be a further barrier to achieving biofilm-active concentrations ^7^. Among *S. aureus*, the involvement of methicillin resistant *S. aureus* (MRSA) in these infections, with reported rates of up to 46 % in the USA ^8^, further limits therapeutic options and often results in longer clinical courses and poorer outcomes. *P. aeruginosa* is resistant to multiple antibiotic classes, both inherently and through acquisition of antibiotic resistance genes. Given the combined challenges of multidrug resistance and biofilm involvement in chronic wound infections, therapeutic agents with mechanisms unrelated to those of conventional antibiotics that can penetrate and kill biofilms are urgently needed.

Structurally nano-engineered antimicrobial peptide polymers (SNAPPs) have received much attention as novel antimicrobials due to their potent antimicrobial activity which targets the cell membrane, unlike most conventional antibiotics. SNAPPs based on repeating units of valine and lysine arranged in a star-shape around a central core developed by Lam *et al.* ^9^ have antimicrobial activity against Gram-negative and Gram-positive bacteria. The authors suggest that the star arrangement affords greater membrane-disrupting capacity than linear forms. For these copolymer SNAPPs, antimicrobial activity increased with respect to the number of star-arms and the star-arm length ^10^. The antimicrobial properties of linear polymers, such as ε- poly(L-lysine) (ε-PLL) have been known for decades and renewed interest in their topical application, for example in wound dressing, is emerging ^11^. Indeed, we have investigated the integration of SNAPP-like star PLL into hydrogels and found potency against *S. aureus* and *E. coli* ^12, 13^.

In this study, we aimed to investigate the antibacterial and anti-biofilm properties of a series of poly(L-lysine) (PLL)-based polymers in star and linear arrangements, against clinically relevant bacteria, *S. aureus* (including MRSA) and *P. aeruginosa* from diabetic foot infections and other wound infections. Importantly, we report highly similar activity of linear and star arrangements of the PLL backbone, which is different to the behaviour reported for linear copolymers of valine and lysine. For PLLs with star-shaped architecture, antimicrobial activity was retained with various hydrophobic amino-acid modifications to the star arms. As such, these peptide-mimetic polymers offer structural flexibility for topical clinical applications and have potential for further development, particularly in the setting of chronic wound infections.

## Methods

### Ethics statement

Ethical approval was not sought as it is not required for research on micro-organisms, which are not considered as belonging to the patient or any individual person. No human tissue was used in this study and no additional patient specimen processing or investigations were undertaken. No patient data was collected, processed or stored. Swabs were taken in 2019 at Beaumont Hospital, Dublin, with the patient’s verbal consent, by healthcare staff, from the wound or base of foot ulcers (for DFW) for clinical investigation, as part of their routine clinical care to support the diagnosis of wound infection/diabetic foot infection. Swabs are subsequently routinely processed for recovery and identification of colonizing/infecting organisms by the Beaumont Hospital Microbiology Laboratory. When either *S. aureus* or *P. aeruginosa* were identified, pure cultures of these bacteria were provided to the researcher. The study bacteria were received entirely anonymously without any associated patient identifier or data. 

### PLL-polymer synthesis

This research used the following:

M03079 - H-Lys(Z)-OH - ε-carbobenzyloxy-L-lysine

M02961 - H-Phe-OH - L-phenylalanine

M03002 - H-lle-OH - L-isoleucine

M03100 - H-Tyr(Bzl)-OH - O-benzyl-L-tyrosine

All chemicals and solvents were obtained from Sigma Aldrich (Ireland) unless otherwise noted. ε-carbobenzyloxy-L-lysine (M03079), L-isoleucine (M03002), L-phenylalanine (M02961) and O-benzyl-L-tyrosine (M03100) were purchased from Fluorochem (UK)

The polypeptides and copolypeptides were synthesised via *N*-carboxyanhydride ring-opening polymerisation (NCA ROP) as previously described ^14^. For synthesis of ε-carbobenzyloxy-L-lysine (ZLL) NCA: ε-carbobenzyloxy-L-lysine (15 g, 53.51 mmol) and α-pinene (147524, 18.22 g, 133.78 mmol) were suspended in 180 mL of dry tetrahydrofuran (THF) and heated under reflux. A solution of triphosgene (330752, 7.15 g, 24.08 mmol) in 30 mL dry THF was added drop-wise to the suspension. The suspension was refluxed until all solids disappeared and the solution became clear (5 hours). The solution was then cooled, filtered and 2/3 of the volume was removed under vacuum. It was then precipitated by addition of 250 mL hexane and stored overnight at -18 °C. The NCA solid was dried, redissolved in dry ethyl acetate and filtered. The NCA solution was then recrystallised thrice in ethyl acetate/hexane (1:1.5) and subsequently washed with hexane to remove any trace impurities. It was vacuum dried to afford a colourless fluffy solid (yield 83%). The NCAs of L-isoleucine (LI), L-phenylalanine (LP) and O-benzyl-L-tyrosine (BLT) were synthesised using the same method. For synthesis of 8arm-star-poly(L-lysine) (G2(8)PLL _20_) – (representative procedure): ε-carbobenzyloxy-L-lysine (ZLL) NCA (1 g, 2.27 mmol) was dissolved in 15 mL CHCl _3_ and 3 mL dimethylformamide. G2 poly(propylene imine) (PPI) dendrimer (15.87 mg, 2.04 × 10 ^-2^ mmol) was dissolved in 3 mL of CHCl _3_ and it was then added quickly to the NCA solution. The solution was allowed to stir until Fourier-transform infrared spectroscopy analysis confirmed total consumption of the ZLL NCA monomer. The polymer was then precipitated into excess diethyl ether and dried under vacuum in a dessicator (yield 85%). To deprotect the polypeptide, 500 mg of G2(8)PLL _20_ was dissolved in 10 mL of trifluoroacetic acid (TFA), followed by dropwise addition of 3 mL of 33 wt% hydrobromic acid (HBr) in acetic acid. The solution was stirred for 18 hours followed by precipitation into excess diethyl ether (150 mL). After drying in vacuo, it was dissolved in deionised water and loaded into a 3500 molecular weight cut off (MWCO) membrane (Thermofisher Scientific, Ireland). Dialysis was performed against deionised water for four days with frequent water replacement, followed by lyophilisation (yield 72%). The other polypepft-irtides were prepared in the same way, using different PPI dendrimers to form G3 and G4 PLL derivatives respectively. The addition of NCA comonomers (LI NCA, LP NCA or BLT NCA) along with ZLL NCA using the same method afforded the statistical copolypeptides containing hydrophobic amino acid repeat units. In this way, three star PLLs were generated: G2(8)PLL _20_ (8-star-PLL) (G2 = generation 2 poly(propylene imine) dendrimer (PPI) core, (8) = 8 polymeric L-lysine arms with 20 L-lysine subunits per arm, *M _n_* = 21,400 g/mol); G3(16) PLL _10_ (16-star-PLL) (G3 = generation 3 PPI core, (16) = 16 polymeric L-lysine arms with 10 L-lysine subunits per arm, *M _n_* = 22,300 g/mol); and G4(32)PLL _5_ (32-star-PLL) (G4 = generation 4 PPI core, (32) = 32 polymeric L-lysine arms with 5 L-lysine subunits per arm, *M _n_* = 24,200 g/mol). The equivalent linear polymer PLL _160 _was a comparator for this series (no dendrimer core) *M _n_* = 20,600 g/mol. A further G3(16)PLL _20_ series was generated with an additional five hydrophobic amino acid residues attached to the star arms as follows: G3(16)PLL _20_-co-PLI _5 _(G3=generation 3 PPI core, 16 = polymeric L-lysine arms, PLL20 = 20 poly(L-lysine) per arm, co-PLI _5 _= 5 poly (L-isoleucine) per arm, *M _n_* = 50,300 g/mol, G3(16)PLL _20_-co-PLP _5_, co-PLP _5 _= 5 poly (L-phenylalanine) per arm, *M _n_* = 53,000 g/mol and G3(16)PLL _20_-co-PLT _5,_ co-PLT _5_= 5 poly (L-tyrosine) units per arm, *M _n_* = 54,300 g/mol.

### Bacterial strains and isolates

A laboratory strain from the American Type Culture Collection, *S. aureus* ATCC 25923, and the widely used *P. aeruginosa* strain PAO1 (ATCC 156920) which originates from a wound infection ^15^ were used to establish assay conditions for antimicrobial evaluation of polymers. Two collections of clinical isolates were further evaluated. One consisted of *S. aureus* and *P. aeruginosa* recovered from swabs taken as part of routine clinical care from patients with suspected infection during monthly multidisciplinary diabetic foot clinics taking place in Beaumont Hospital, Dublin in 2019 for microbiological diagnosis. The second consisted of 10 *S. aureus*, five of which were MRSA, which were from confirmed wound infections from the same hospital in the same collection period (2019). All swabs were processed by the Beaumont Hospital Microbiology Laboratory, Dublin and were provided anonymized, as pure cultures. Organism identity was confirmed by matrix-assisted laser desorption/ionization- time-of-flight mass spectrometry (MALDI-TOF) using a MALDI Biotyper (Brüker). MRSA was confirmed by the growth of pink colonies on MRSASelect ^TM^ agar (BioRad, UK). In total 30 *S. aureus* (of which 10 were MRSA) and five *P. aeruginosa* were collected. For testing novel antimicrobials, clinical isolates were selected randomly from those available to the researchers with numbers chosen as a cross-section, representative of those colonizing and infecting DFIs. The lower numbers of *P. aeruginosa* available compared to *S. aureus* recovered are also representative of the trends in recovered pathogens. Within the isolate collection, for determination of statistical significance, e.g. comparison of the activity of linear with star PLLs, biofilm formation, greater than three clinical isolates in each group was considered appropriate.

### Bactericidal assay

Assays were performed as described by Forde *et al.* ^16^ with modifications. Briefly, isolated colonies from overnight growth of *S. aureus* on Columbia Blood Agar (CBA) or *P. aeruginosa* on MacConkey agar were suspended in PBS to the density of 0.5 MacFarland Standard using a Densichek Meter (approximately 5 × 10 ^7^ colony forming units (CFU)/ml). Suspensions were further diluted in the assay buffer (10mM potassium phosphate buffer, pH7.4, containing 0.2% w/v bovine serum albumin (BSA)). Assays contained approximately 10 ^5 ^CFU/ml bacteria and varying concentrations of polymers (0.001μM-10μM) in a total volume of 100μl of assay buffer. Following incubation at 37°C for 1 hour, 900 μl of NaCl (0.95% w/v) was added to assays and 100 μl aliquots were spread onto Mueller Hinton Agar. Plates were incubated at 37°C overnight in a static incubator and resulting colonies were counted. Where numbers were ‘too numerous to count’, further dilutions were plated. Killing activity was determined based on CFU/ml from assays containing PLL polymers (log ^10^ reduction) compared to CFU/ml on control plates containing no polymer. Rifampicin or gentamicin were used as comparators where appropriate against *S. aureus* and *P. aeruginosa*, respectively.

### Biofilm viability assay

Effects of the polymers on biofilms were investigated as described previously ^17^. Colonies from overnight cultures were isolated and used to prepare suspensions to the density of a 0.5 McFarland standard using a Densichek meter (bioMérieux, Ireland). The suspensions were further diluted 1/100 in tryptic soy broth (TSB) containing 0.5% glucose and 100 μL added to 96 well polystyrene plates. Plates were incubated for 24 hours at 37°C. Following washing (3 times with distilled water), the biofilms were treated with various PLL polymers (50 μM) by the addition of 100 μL to wells. Rifampicin or gentamicin (100 μM) was added to biofilm wells as comparators and 100 μl of KPB, pH 7.4 was added to biofilm wells as controls. Wells containing TSB only were included as media controls. The treated plates were incubated at 37°C for 30 minutes. For biofilm viability measurement after treatment, a stock solution of the non-fluorescent redox dye resazurin at 440 μM was diluted 1/5 in TSB and 100 µL was added to each well. Plates were incubated at 37°C for 1 hour in the dark. Fluorescence at 544 nm excitation and 590 nm emission was measured using a Perkin Elmer 2030 Multi-label Reader Viktor X3 spectrophotometer.

## Data analysis

Data were analysed and graphs constructed using Microsoft Excel 2016. The log _10_CFU/ml was determined in controls and treated assays and used to determine the mean log _10 _reduction in CFU/ml for each PLL structure or antibiotic. For anti-biofilm assays, mean fluorescence intensity units were determined in control and treated assays. Tests of statistical significance of means between groups (control and treated) were by unpaired t-tests using GraphPad Quickcalcs on-line software.

## Results

### Star and linear polypeptide structures can be synthesised

**Figure 1.**
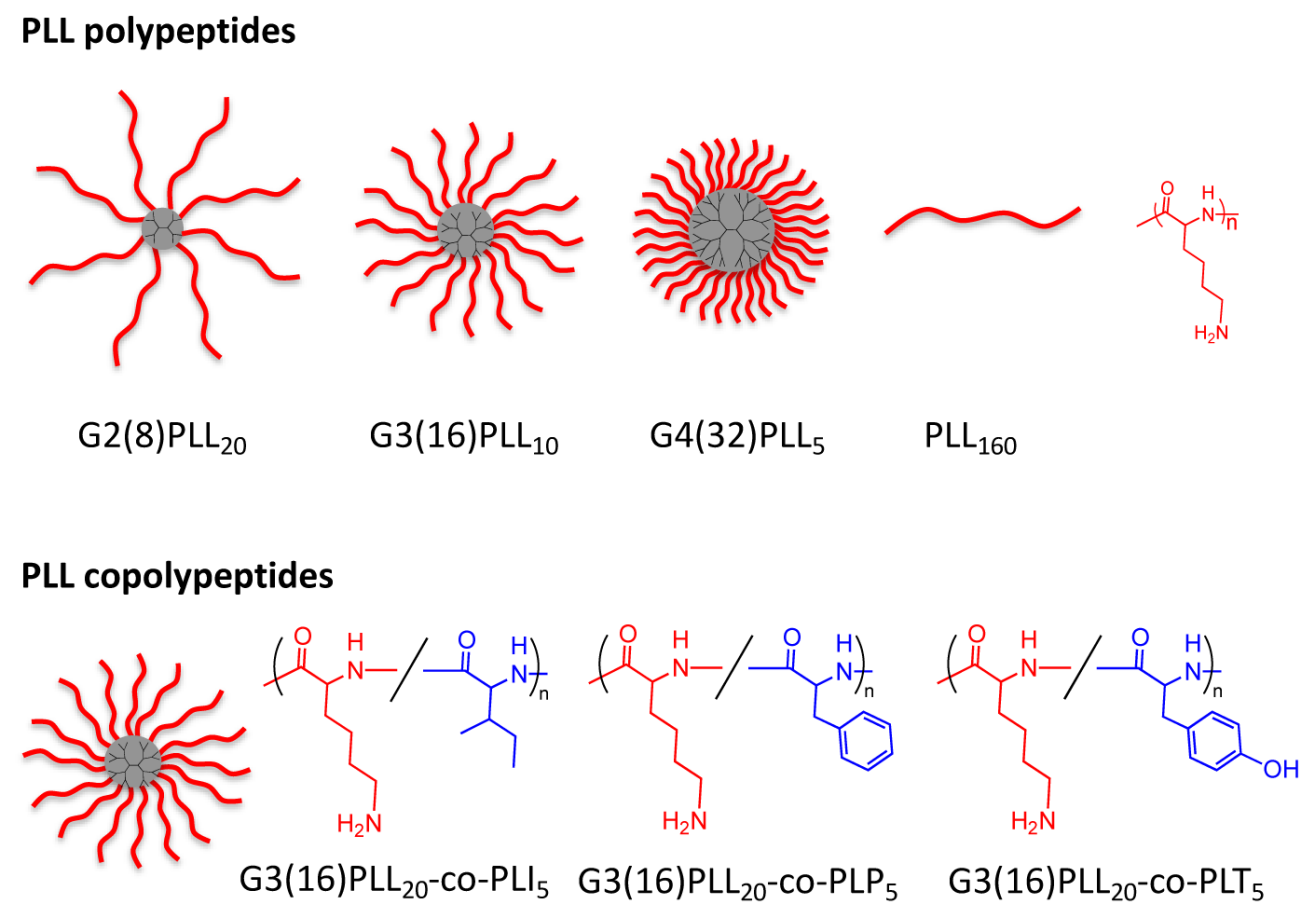
Structures of star poly(L-lysine) homo- and copolypeptides.

A set of star-shaped poly(L-lysine) structures with 8, 16 and 32 arms were synthesised by the ring-opening polymerisation of ε-carbobenzyloxy-L(lysine) *N*-carboxyanhydride (ZLL NCA), followed by subsequent deprotection and purification ^18, 19^. The total number of PLL units was kept constant at 160 for all star polypeptides resulting in decreasing arm length with increasing number of arms ( Figure 1). A linear PLL was also obtained. In addition, 16-arm statistical copolypeptides comprising 20 repeating units of PLL and 5 repeating units of different hydrophobic amino acids were synthesised. The latter were selected from isoleucine, phenylalanine and tyrosine. These and similar structures were previously investigated extensively as delivery tools for applications in tissue engineering where their versatility in delivering therapeutic cargos into mesenchymal stem cells (MSC) has been demonstrated ^14^.

### Linear PLL polymer has similar antimicrobial activity to equivalent PLLs with star-shaped architecture

**Figure 2.**
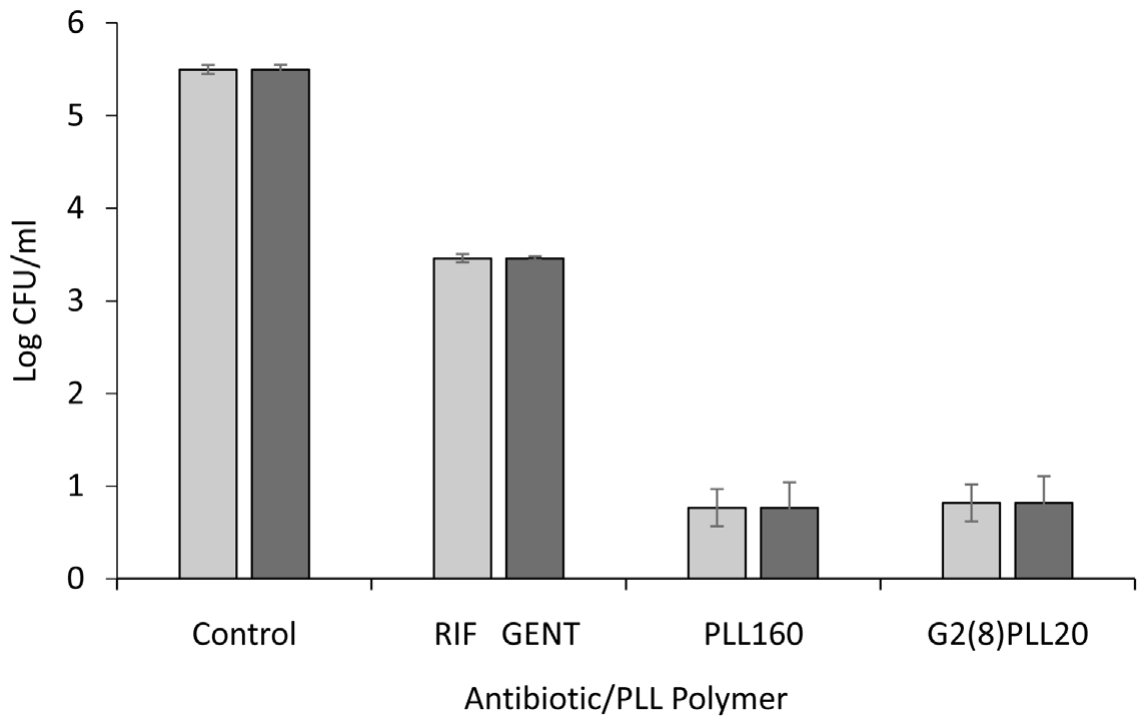
Bactericidal activity of poly-L-lysine polymers against *S. aureus* and *P. aeruginosa* laboratory strains compared to antibiotics. Log CFU/ml for a fixed concentration of bacteria following incubation with 10μM PLL polymers in 10mM potassium phosphate buffer (1 hour, 37 ^o^C), 0.2% BSA. *S. aureus* ATCC 25923 (light grey bars), *P. aeruginosa* PAO1 (dark grey bars). Rifampicin (RIF) and gentamicin (GENT) were used at 10 μM for comparison. Data shown are the mean ± SEM of three assays carried out in duplicate.

**Figure 3.**
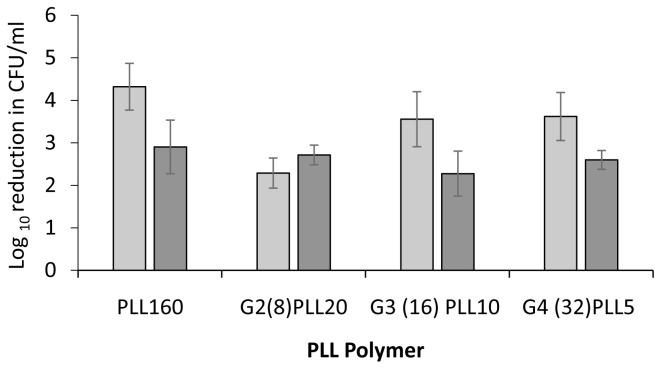
Bactericidal activity of poly-L-lysine polymers against *S. aureus* and *P. aeruginosa* laboratory strains. Log reduction in CFU/ml for a fixed concentration of bacteria following incubation with 1μM PLL polymers in 10mM potassium phosphate buffer (1 hour, 37 ^o^C), 0.2% BSA. *S. aureus* ATCC 25923 (light grey bars), *P. aeruginosa* PAO1 (dark grey bars). Data shown are the mean ± SEM of three assays carried out in duplicate.

Potent bactericidal activity was demonstrated against the reference *S. aureus* strain ATCC 25923 and *P. aeruginosa* strain PAO1. Preliminary testing of 10 μM PLLs, linear (PLL _160_) Vs star architecture (G2(8)PLL _20_) for 1 hour against these bacteria resulted in killing greater than the upper limit of the assay (log 5) compared to log 2 reduction in CFU/ml using the antibiotics rifampicin or gentamicin ( Figure 2). A lower concentration of 1 μM was therefore investigated to observe differential activity among PLLs. Incubation with PLL-polymers at 1 μM for 1 hour resulted in a 2-4 log reduction in CFU/ml for *S. aureus* with the greatest activity for the linear PLL _160_ (4.23 log) and the least for the star architecture with 8 arms around the central core (G2(8)PLL _20_ = 2.29 log). Improved activity was found as the arm numbers increased to 16 and 32. At 1 μM, 2-3 log reduction in CFU/ml was found for PAO1 with little difference in activity with linear or any of the star architectures, in terms of number of star arms (8, 16, 32) or length of star arms ( Figure 3).

### Antimicrobial activity is retained on addition of hydrophobic amino acids to the star architecture

**Figure 4.**
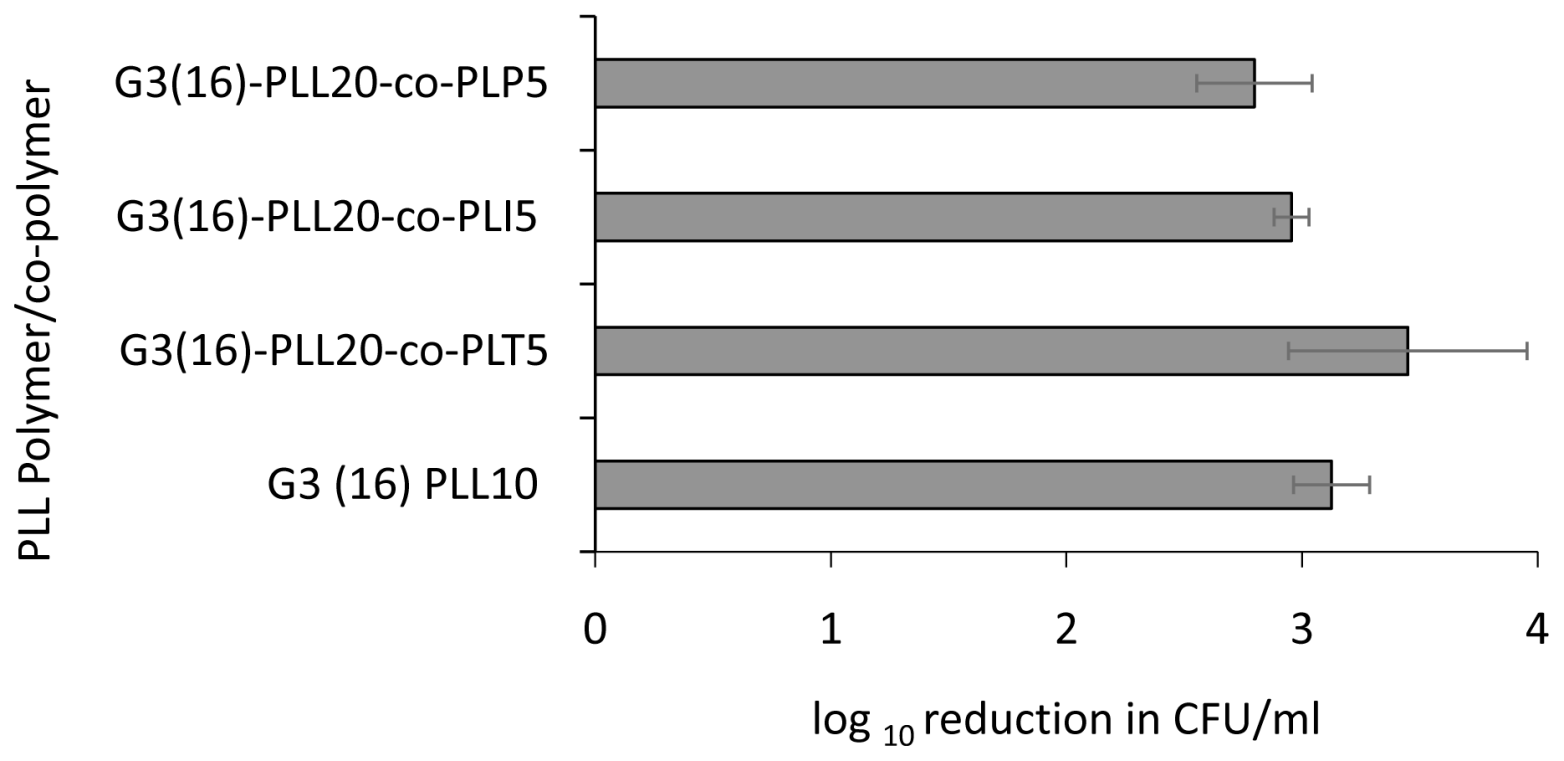
Comparative bactericidal activity of poly-L-lysine G3(16) copolymers series with hydrophobic amino acid isoleucine, tyrosine and phenylalanine. Log reduction in CFU/ml with respect to control for a fixed concentration of *S. aureus* ATCC 25923, following incubation with 1μM polymer/copolymer in 10mM potassium phosphate buffer (1 hour, 37 ^o^C), 0.2% BSA. Data shown are the mean ± SEM of three assays carried out in duplicate.

Using the G3(16)PLL _20_ series as a model, further modification of the star arms by the addition of copolypeptides of the amino acids tyrosine, isoleucine or phenylalanine to the poly-lysine arms were made. These modifications did not significantly change the antimicrobial activity against *S. aureus* ( *p* value ≥ 0.25) which remained at 3-4 log following 1 hour incubation with 1 μM ( Figure 4).

### Clinical isolates from wound infections and DFW are susceptible to poly(L-lysine) polymers

**Figure 5.**
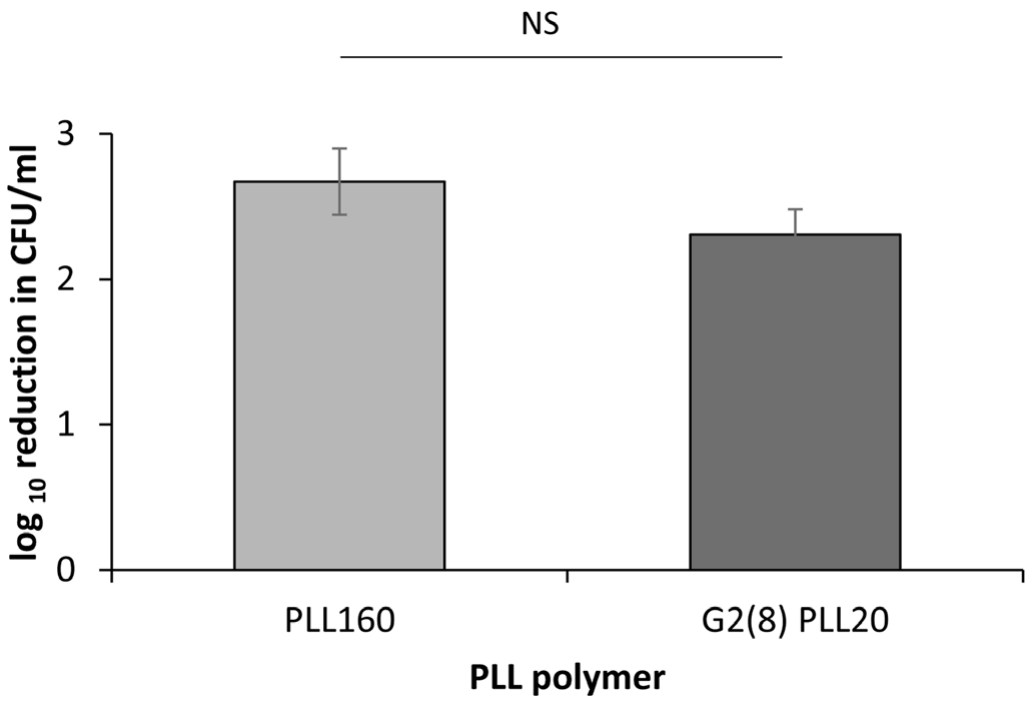
Comparison of Bactericidal activity of poly-L-lysine polymers, PLL _160_ and G2(8)PLL _20_ against *S. aureus* clinical isolates from wound infections. Log reduction in CFU/ml for a fixed concentration of bacteria following incubation with 1μM PLL or G2(8)PLL _20_ polymer in 10mM potassium phosphate buffer (1 hour, 37 ^o^C), 0.2% BSA compared to control (no treatment). Assays carried out in duplicate on three occasions for n=10 *S. aureus* clinical isolates. NS = not statistically significant, p > 0.1 by unpaired t-test.

**Figure 6.**
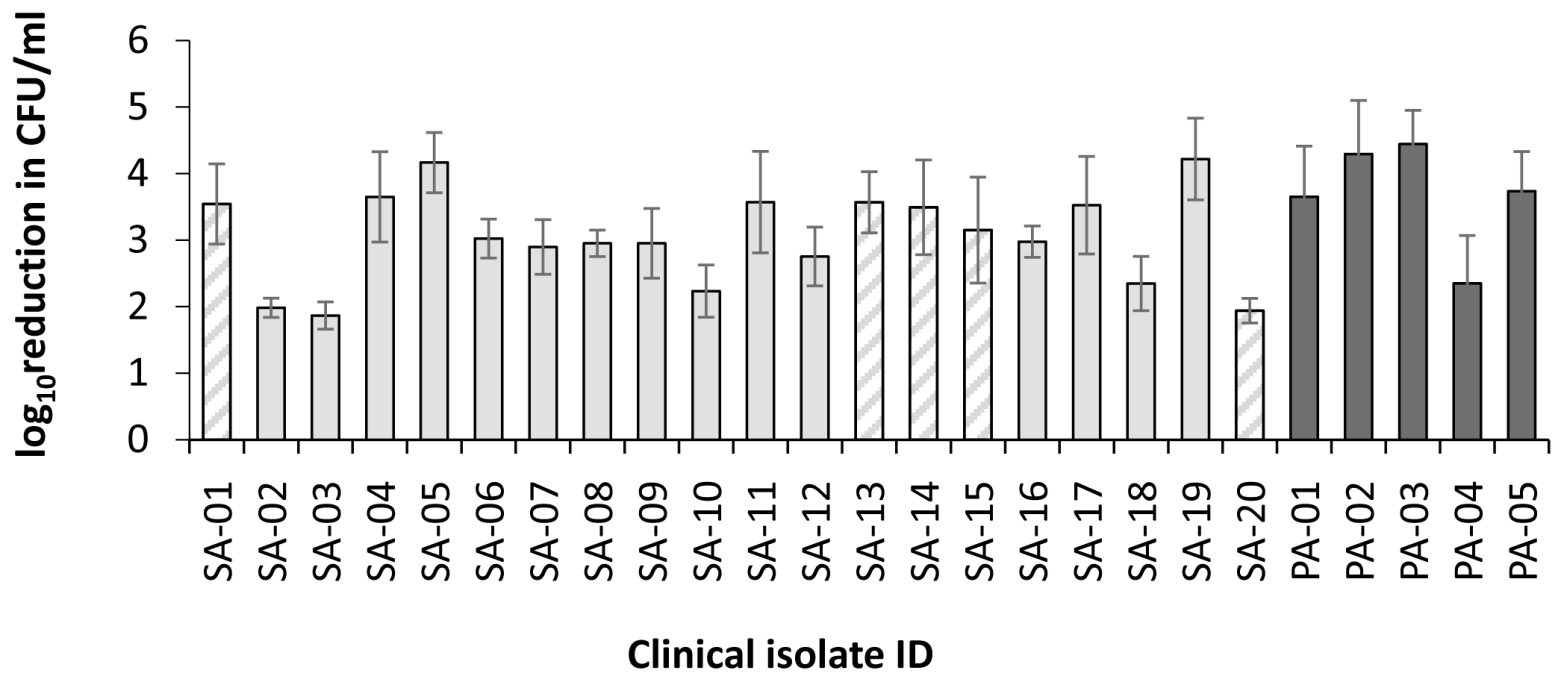
Bactericidal activity of linear PLL _160_ against *S. aureus* and *P. aeruginosa* isolates from suspected diabetic foot infections. Log reduction in CFU/ml for a fixed concentration of bacteria following incubation with 1 μM PLL _160_ in 10mM potassium phosphate buffer (1 hour, 37 ^o^C), 0.2% BSA compared to control (no treatment). Data shown are the mean ± SEM for assays carried out in duplicate on three occasions per isolate. Hatched bars indicate MRSA isolates.

Clinical isolates recovered from colonized wounds and infections often have different behaviours and properties to laboratory strains. Using PLL _160 _and G2(8)PLL _20_ again at 1 μM to reveal potential potency differentials among isolates, both linear and star PLLs remained active against *S. aureus* (n=10 isolates including 5 MRSA) recovered from confirmed wound infections with mean values of 2.3 and 2.7 log reduction in CFU/ml respectively ( Figure 5). As the linear PLL-polymer was the most active at this lower concentration, it was selected for investigation across a further collection of clinical isolates from DFW/DFI. Potent bactericidal activity was observed for *S. aureus* and *P. aeruginosa* clinical isolates recovered from DFW/DFI with some variability between isolates ( Figure 6). For *S. aureus*, the mean log reduction was 3.04 ± 0.16 (range 1.86 to 4.22, n=20), for *P. aeruginosa* isolates the mean log reduction was 3.96 ± 0.82 (range 2.35 to 4.29, n=5).

### Anti-biofilm activity of PLL-polymers

**Figure 7.**
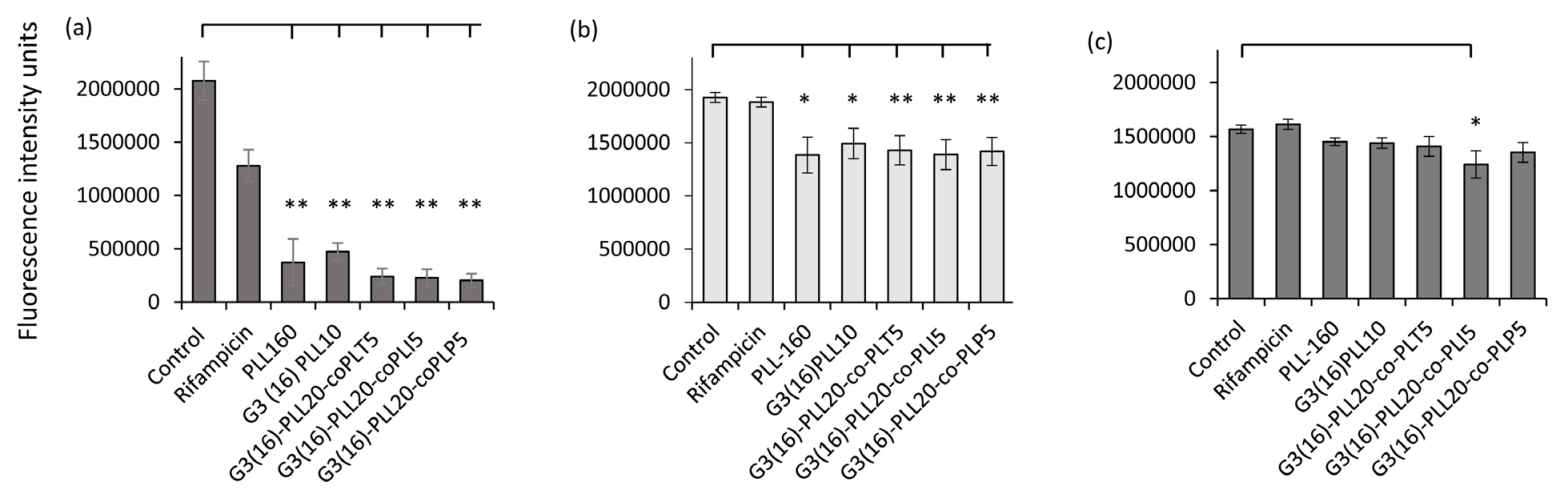
Investigation of PLL polymer-induced loss of biofilm viability by resazurin staining of 24 h biofilms. *S. aureus* reference strain ATCC 25923 ( **a**) and *S. aureus* DFW/DFI clinical isolates (n=10) ( **b**) or *P. aeruginosa* DFW/DFI clinical isolates (n=5) recovered from diabetic foot infections. Biofilms were treated with each polymer (50 μM) for 30 minutes at 37 ^o^C using at least six replicates per assay. Data shown are the mean ± SEM. Rifampicin/gentamicin concentration = 100 μM, * p ≤ 0.05, ** p ≤ 0.01.

The G3(16)PLL _20_ series, including copolymer modifications of the star arms showed statistically significant reduction in biofilm viability over a short time of exposure (30 minutes) to biofilms of *S. aureus* clinical isolates ( *p* ≤ 0.05 (linear and star G3(16)PLL _20 _or *p* ≤ 0.01, co-polymer series). However, only one candidate, G3(16)PLL _20_-co-PLI _5 _had anti-biofilm activity against *P. aeruginosa* isolates ( Figure 7). Rifampicin and gentamicin in comparison demonstrated negligible anti-biofilm activity *in-vitro* under these conditions.

## Discussion

The potential antimicrobial features of star-shaped peptide mimetic polymers has gained much interest since the discovery by Lam *et al.* ^9^ of low-cost, copolypeptides of lysine and valine synthesised by poly(amido amine) (PAMAM) dendrimer initiated NCA ROP of their corresponding monomers. Those structures have been extensively studied, revealing many features that are critical for their development as alternative antimicrobials, including high potency, low toxicity, activity in *in-vivo*-like physiological conditions and independent of bacterial antibiotic resistance mechanisms ^20^. Here we report a further family of SNAPPs, Star-PLLs, developed originally as biocompatible polymeric nano-carriers for drug-delivery ^14, 21^ applications, as having both antimicrobial and anti-biofilm activity against bacteria recovered from DFW and DFI. Interestingly, in addition, we found the linear equivalent PLL to have similar activity to star-forms.

 Diabetic foot infection is the commonest cause of hospitalisation among those with diabetes and the complexity of management may result in lengthened hospital stays. Therefore, to ensure better clinical outcomes for patients, there is a critical need to more effectively manage and treat these infections. A systematic review of antimicrobial agents for chronic wounds, including DFWs, concluded that few systemic agents improved outcomes, but faster healing rates were achieved with additional use of several topical substances ^4^. Synthetic antimicrobial peptides, such as pexiganan, show broad-spectrum activity against DFI pathogens with "all organism" MIC _90 _of 32 μg/ml reported ^22^. Notably, pexiganan cream used alone was similar in effectiveness to a systemic antibiotic (ofloxacin) in the treatment of mildly infected diabetic foot ^23^. Peptide mimetic polymers with potent bactericidal activity against DFI pathogens, as shown here, may offer enhanced properties for topical wound treatment based on their biocompatible properties which have emerged from studies of their potential in nanocarrier/drug delivery applications.

In the present study, antimicrobial activity was maintained for all PLL polymers investigated with varying architectures from linear to star-shaped, and some with specific copolymer modifications. For all structures, activity was superior to comparator antibiotics, rifampicin or gentamicin, under the same assay conditions. In terms of structure activity relationships (SAR), bactericidal activity was greater when arm number was increased from 8 to 16 but no further increase was noted when further increased to 32. Notably, in this SNAPP series, to maintain equivalence in the number of lysine residues overall, for comparison to the linear polymer, the arm length was decreased (from 20 to 5) with increasing arm number (from 8 to 32). SAR has been studied for a copolymer series of SNAPPs by Shirbin *et al.* who report antimicrobial activity proportional to the number and length of star arms, albeit with different amino-acid structures. Interestingly, our finding of equivalent antimicrobial activity irrespective of the linear *Vs* star architecture is markedly different to that reported for star copolymers of L-lysine and L-valine against reference ATCC strains ^9^. Lam *et al.* reported superior activity of star polymers compared to linear, while, for *S. aureus* reference strain ATCC25923, we found superior activity of linear PLL _160_ which was statistically significant ( *p* value ≤ 0.05) compared to the equivalent star 8-mer PLL (G2(8)PLL _20_). Furthermore, when tested against clinical isolates of *S. aureus* the bactericidal activity was similar for linear and star PLLs ( Figure 5) although inter-isolate variability in activity (PLL _160_) was evident ( Figure 6). As previously highlighted by us, reference strains are not always representative of patient isolates and reliance on reference strains alone may over- or underestimate activity ^24^.

Biofilm production by infecting bacteria is a significant contributor to poorer healing rates and clinical outcomes for patients with wounds infections. All PLL polymers reduced the viability of *S. aureus* biofilm and this was independent of PLL architectures or modifications. However biofilms of *P. aeruginosa* were less susceptible to PLLs with only one co-polymer (G3(16)PLL _20_-co-PLI _5_) showing statistically significant reduction in metabolic activity. While moderate anti-biofilm activity was found against clinical isolates, the activity against *S. aureus* reference strain ATCC25923 was significantly greater for all polymers. This finding highlights the need for caution in using reference strains to represent antimicrobial properties against clinical isolates. The structure and physiological properties of biofilms formed by clinical isolates can vary widely under laboratory conditions and even more so when influenced by the *in-vivo* environment of infection ^25^. The effect of these differences on their response to novel antibiotics and novel antimicrobials should be considered when testing novel anti-biofilm agents. 

Our study has limitations. Bactericidal assays were performed in a specific buffer, similar to other published studies and representing physiological conditions (pH 7.4, 10mM potassium phosphate, 37 ^o^C). Varying these conditions may alter or reduce activity as has been shown by others ^20^. Further investigation of bactericidal activity under conditions that mimic the pathological environment of wound sites would be important in further development for this clinical application, such as the addition of human serum/wound exudate. Nonetheless, the maintenance of potent activity across clinically relevant isolates, recovered from wounds, demonstrates the antimicrobial potential of this approach. We used an indirect measure of biofilm viability based on metabolic activity rather than viable plate counts and only 24 hour biofilms were tested, showing only moderate to low anti-biofilm activity for this PLL-series against clinically relevant isolates. Improving the anti-biofilm properties of PLL-based and other polymers may be possible iteratively with further structural modifications and should focus on both removal and killing of mature biofilms. As expected based on microbial aetiology, fewer *P. aeruginosa* clinical isolates were recovered from DFW/DFI compared to *S. aureus* during the collection period and therefore *P. aeruginosa* data represent more limited numbers.

Recently Star-PLL, including G4(32)PLL _40_, have been used to functionalise collagen-based scaffolds with DNA-based therapeutic cargos for bone regeneration/repair applications (bone-morphogenetic protein-2 plasmid (pBMP-2) and vascular endothelial growth factor plasmid (pVEGF)). Osteogenic differentiation of mesenchymal stem cells (MSCs) was demonstrated *in-vitro* using the PLL-platform and studies in a rat model showed accelerated healing of bone defects *in vivo* ^26^. The sequelae of diabetic foot wounds and infections may involve abnormal structural defects, e.g. Charcot foot, hammer toe which may further facilitate infection progression ^27^. Adding our finding of bactericidal/anti-biofilm activity relevant specifically to diabetic foot infections, to the properties of these PLL-based biocompatible structures, suggests enhanced and multimodal applications in this setting which will be further explored.

## Data availability statement

### Underlying data

Zenodo: Modified poly(L-lysine)-based structures as novel antimicrobials for diabetic foot infections, an in-vitro study. https://doi.org/10.5281/zenodo.5537160

This project contains the following files:

- Figure 2_PLL_AntimicrobialDFH21V2.xlsx: Bactericidal Activity against *S. aureus* ATCC25923 and *P. aeruginosa* PAO1]
- Figure 3_PLL_AntimicrobialDFH21V2.xlsx: Bactericidal activity of poly-l-lysine polymers against *S. aureus* and *P. aeruginosa*
- Figure 4_PLL_AntimicrobialDFH21V2.xlsx: Bactericidal activity of poly-L-lysine G3(16) copolymers series with hydrophobic amino acid isoleucine, tyrosine and phenylalanine
- Figure 5_PLL_AntimicrobialDFH21V2.xlsx: Comparison of bactericidal activity of poly-l-lysine polymers, PLL160 and G2(8))PLL20 against *S. aureus* clinical isolates from wound infections
- Figure 6_PLL_AntimicrobialDFH21V2.xlsx: Bactericidal activity against *S. aureus* and *P. aeruginosa* clinical isolates from diabetic foot wounds
- Figure 7_PLL_AntimicrobialDFH21V2.xlsx: Antibiofilm activity against *S. aureus* ATCC25923, clinical isolates of *S. aureus* and *P. aeruginosa* from wounds

Data are available under the terms of the Creative Commons Attribution 4.0 International license (CC-BY 4.0).
