# Supplementary material for: Modified poly(L-lysine)-based structures as novel antimicrobials for diabetic foot infections, an in-vitro study
Source: HRB Open Res. 2022 Jan 12;5:4. [Version 1] doi: 10.12688/hrbopenres.13380.1 (PMC9366240; doi:10.12688/hrbopenres.13380.1)
Supplement: Peer review history

# Peer review: Reviewer response for version 1

**DOI:** 10.21956/hrbopenres.14574.r32553

**Published:** 2022-08-10

**Authors:** Thokur Sreepathy Murali

**Affiliations:** 1 Department of Biotechnology, Manipal School of Life Sciences, Manipal Academy of Higher Education, Manipal, Karnataka, India

In the manuscript entitled "Modified poly(L-lysine)-based structures as novel antimicrobials for diabetic foot infections, an *in-vitro* study", the authors have evaluated the antimicrobial and anti-biofilm activity of linear and star-shaped poly(L-lysine) polymers against clinical isolates of *S. aureus* and *P. aeruginosa* obtained from diabetic foot wounds. They found potent bactericidal activity and slighlty better antibiofilm activity compared to other antibiotics. Based on these preliminary data, the authors conclude that the polypeptides can be used by topical application in treating chronic wound infections. The manuscript is well written and provides the methods to reproduce the findings. The discussion section provides a summary and importance of their findings with support of relevant literature. The authors also have provided a list of shortcomings of their study. The authors can comment on the stability of these polypeptides in wound environment which are known to be rich in peptidases. I have also listed a few minor comments below:

- PLL-polymer synthesis: "The other polypepft-irtides were prepared in the same way," - Please correct
- "The lower numbers of *P. aeruginosa* available compared to *S. aureus* recovered are also representative of the trends in recovered pathogens." - Please elaborate on this statement to make it clearer to the audience. 
- Figure 4: The error bars in experiments with tyrosine as copolymer is slightly higher. Any specific reason? 
- Antibiofilm activity: "However, only one candidate, G3(16)PLL _20_-co-PLI _5 _had significant anti-biofilm activity against *P. aeruginosa* isolates ( Figure 7)." Also, the legend for antibiotic used against *P. aeruginosa* clinical strains is mentioned as Rifampicin. Or is it gentamicin? Further, the effect of gentamicin on PA01 is not shown. 

In summary, I recommend that the manuscript can be accepted for indexing.

Is the work clearly and accurately presented and does it cite the current literature?

Yes

If applicable, is the statistical analysis and its interpretation appropriate?

Yes

Are all the source data underlying the results available to ensure full reproducibility?

Yes

Is the study design appropriate and is the work technically sound?

Yes

Are the conclusions drawn adequately supported by the results?

Yes

Are sufficient details of methods and analysis provided to allow replication by others?

Yes

Reviewer Expertise:

Microbiome of diabetic foot ulcer infections

I confirm that I have read this submission and believe that I have an appropriate level of expertise to confirm that it is of an acceptable scientific standard.

# Peer review: Reviewer response for version 1

**DOI:** 10.21956/hrbopenres.14574.r32428

**Published:** 2022-07-05

**Authors:** Daojun Liu

**Affiliations:** 1 Department of Pharmacy, Shantou University Medical College, Shantou, China

The authors conducted a concise but comprehensive study to compare in vitro antimicrobial performance of linear and star-shaped PLL against DF infections. The results are very interesting and have great reference value. The manuscript can be indexed after minor revision if the following questions are carefully addressed.

1. Remove “novel” from the title of the manuscript, the description of “diabetic foot infections” in the title is also misleading because *in vivo* experiments are not involved.
2. The characterization of all polymers such as NMR and GPC measurements should be provided.
3. For the hydrophobic amino acid-modified polypeptides, please clarify whether the arm is a block copolypeptide or a random copolypeptide.
4. For the antimicrobial study, the determination of MIC or MBC values is somewhat obligatory.
5. The different antimicrobial performance of PLL against reference strains and clinical isolates might be explained or speculated.
6. In Figure 7, the item (c) is missing.

Is the work clearly and accurately presented and does it cite the current literature?

Yes

If applicable, is the statistical analysis and its interpretation appropriate?

Yes

Are all the source data underlying the results available to ensure full reproducibility?

Partly

Is the study design appropriate and is the work technically sound?

Yes

Are the conclusions drawn adequately supported by the results?

Yes

Are sufficient details of methods and analysis provided to allow replication by others?

Partly

Reviewer Expertise:

Antimicrobial and antitumor polypeptides

I confirm that I have read this submission and believe that I have an appropriate level of expertise to confirm that it is of an acceptable scientific standard.
